# Estimation of Undiagnosed *Naegleria fowleri* Primary Amebic Meningoencephalitis, United States^1^

**DOI:** 10.3201/eid2401.170545

**Published:** 2018-01

**Authors:** Almea Matanock, Jason M. Mehal, Lindy Liu, Diana M. Blau, Jennifer R. Cope

**Affiliations:** Centers for Disease Control and Prevention, Atlanta, Georgia, USA

**Keywords:** neuroinfections, primary amebic meningoencephalitis, parasites, *Naegleria* *fowleri*, primary ameba, amebic meningitis, PAM, international classification of diseases, waterborne infections, United States

## Abstract

Primary amebic meningoencephalitis is an acute, rare, typically fatal disease. We used epidemiologic risk factors and multiple cause-of-death mortality data to estimate the number of deaths that fit the typical pattern for primary amebic meningoencephalitis; we estimated an annual average of 16 deaths (8 male, 8 female) in the United States.

*Naegleria fowleri* causes primary amebic meningoencephalitis (PAM); 0–8 laboratory confirmed cases per year are documented in the United States) (*1*). PAM causes <0.5% of diagnosed encephalitis deaths in the United States (*2*). Laboratory-confirmed PAM case-patients in the United States are a median age of 12 years and are identified primarily in southern states during July–September, and 79% are male (*1*,*3*). Many case-patients are identified postmortem; 4 known survivors have been reported in the United States (*1*,*4*). The signs and symptoms of PAM can be mistaken for other more common neuroinfections, such as bacterial meningitis and viral encephalitis (*1*,*4*). Because more than half of neuroinfectious deaths are unspecified (*2*), clinical expertise and diagnostic testing availability are limited, and true PAM incidence is unknown, concern is reasonable that PAM cases might not be diagnosed. In this study, we estimate the magnitude of potentially undiagnosed cases of PAM by applying previously identified epidemiologic risk factors to unspecified neuroinfectious deaths.

We created a list of codes from the International Classification of Disease, 10th revision (ICD-10), for unspecified possible neuroinfectious deaths by using previously published data (*2*), ICD-10 codes from death certificates of known PAM case-patients, and expert opinion. We selected codes from any location on the death record, not strictly the primary or immediate cause of death (http://www.cdc.gov/nchs/deaths.htm). We chose to start in 1999 when death certificate data were first coded by using ICD-10 and ended in 2010, using the most updated data at the time of this analysis. Persons 2–22 years of age were included (±10 years from the average age of 12 years), excluding infants and older adults, who are more susceptible to bacterial meningitis. We applied known risk factors for PAM: 1) geographic location, i.e., states that reported diagnosed cases as of 2010; 2) summer seasons; and 3) sex (*3*). Within this narrowed subset of unspecified neuroinfectious deaths, we reviewed associated ICD-10 codes and removed death records that had more definitive diagnoses.

During 1999–2010, there were 1,676 unspecified neuroinfectious disease deaths among persons 2–22 years old; 49% (826/1,676) occurred during July–September of each year studied, and of those, 23% (192/826) were reported from an included state in the southern United States; 52% (100/192) were male and 48% (92/192) female. An average of 16 (8 male, 8 female) unspecified neuroinfectious deaths per year fit the typical pattern of PAM, in addition to the average 3 laboratory-confirmed cases annually during this time period. 

Among all unspecified neuroinfectious deaths, the most common unspecified neuroinfectious death code used was G03.9 meningitis unspecified (n = 505) (Table). For the top 5 codes, 8%–16% of cases matched all the risk factors and 2 had a sex ratio of exactly 50%. We did not have access to death certificates for 20 known laboratory-confirmed case-patients to determine what ICD-10 codes were used in these cases.

**Table:** Unspecified neuroinfectious death ICD-10 codes by epidemiologic risk factor for primary amebic meningoencephalitis among persons 2–22 years of age, United States, 1999–2010*†

ICD-10 code, disease	Total	In high-incidence states‡ (%)	July–Sept (%)	In high-incidence states,‡ July–Sept

Male patients (%)	Female patients (%)
G03.9, Meningitis unspecified	505	257 (51)	96 (19)	28 (6)	28 (6)
G04.9, Encephalitis, myelitis and encephalomyelitis, unspecified	479	222 (46)	135 (28)	33 (7)	26 (5)
R29.8, Other and unspecified symptoms and signs involving the nervous and musculoskeletal systems	264	112 (42)	63 (24)	8 (3)	13 (5)
G00.9, Bacterial meningitis, unspecified	222	105 (47)	41 (18)	11 (5)	11 (5)
A86, Unspecified viral encephalitis	154	92 (60)	40 (26)	15 (10)	9 (6)
G06.2, Extradural and subdural abscess, unspecified	59	31 (53)	12 (20)	4 (7)	0
A87.9, Viral meningitis, unspecified	38	25 (66)	13 (34)	4 (11)	4 (11)
A89, Unspecified viral infection of the central nervous system	6	2 (33)	3 (50)	0	1 (17)
A83.9, Mosquito-borne viral encephalitis, unspecified	1	1 (100)	1 (100)	0	1 (100)

*Codes without cases (R83.5, A92.9, A85.2, A84.9, A81.9, A94, A06.6) not listed. ICD-10, International Classification of Disease, 10th revision. †The total provided is greater than the total number of cases because each case may have >1 ICD-10 code. ‡Arizona, Arkansas, California, Florida, Georgia, Louisiana, Mississippi, Missouri, Nevada, New Mexico, North Carolina, Oklahoma, South Carolina, Texas, and Virginia.

Our estimate of annual undiagnosed PAM cases shows that unspecified neuroinfectious deaths that fit the epidemiologic pattern of PAM occur infrequently. This estimate likely includes unspecified neuroinfectious death caused by other pathogens. We have no method to differentiate cases that fit the pattern of PAM, but are caused by another pathogen. Bacterial meningitis, which can be mistaken for PAM (*1*), has decreased over approximately the same time period as this study, but does not have the epidemiologic pattern of PAM (*5*). Viral causes (i.e., La Crosse and West Nile viruses) have a similar pattern, occurring during July–September (>80% of cases) and more commonly in males (3:2 male:female ratio) (*6*). An ICD-10 code for West Nile virus, A92.3, was added in 2005. There was only 1 case in our estimate that had the code Mosquito-borne viral encephalitis, unspecified (A83.9). Similar to PAM, cases of arbovirus disease could be included in even less-specific meningitis and encephalitis codes, illustrating that unspecified neuroinfectious deaths are likely caused by several pathogens.

Medical chart review and autopsies, not available for this study, would provide further information about the cause of death. Although this estimate likely captures more than just PAM cases for the reasons we have outlined, it might not capture all potential PAM cases. Reasons for an underestimate include inaccurate ICD-10 coding (*7*) and PAM cases that are outside the typical epidemiologic pattern (e.g., 2 cases in Minnesota [*8*] and out of season, such as adult cases linked to ritual nasal rinsing and sinus irrigation [*9*,*10*]).

Although all available evidence points to PAM being a low-incidence disease in the United States, PAM remains a devastating and nearly universally fatal infection that erodes public confidence in the safety of everyday activities (swimming, using public drinking water) and increased stress on local public health departments that are already overextended. The reports of recent survivors indicate that timely diagnosis and early initiation of anti-amebic therapy may be instrumental in combating this deadly infection (*4*). Therefore awareness, evaluation of risk factors, testing, and early anti-amebic therapy provide the best opportunity for survival (*1*).
